# Injection cessation and relapse to injection and the associated factors among people who inject drugs in Iran: The Rostam study

**DOI:** 10.1186/s13011-023-00583-6

**Published:** 2023-11-29

**Authors:** Soheil Mehmandoost, Ali Mirzazadeh, Mohammad Karamouzian, Mehrdad Khezri, Heidar Sharafi, Armita Shahesmaeili, Saiedeh Haji Maghsoudi, Nima Ghalekhani, Fatemeh Tavakoli, Maliheh Sadat Bazrafshani, Mostafa Shokoohi, Niloufar Aghaali, Ali Akbar Haghdoost, Hamid Sharifi

**Affiliations:** 1 HIV/STI Surveillance Research Center, and WHO Collaborating Center for HIV Surveillance, Institute for Futures Studies in Health, Kerman University of Medical Sciences, Kerman, Iran; 2 Department of Epidemiology and Biostatistics, University of California, San Francisco, CA USA; 3 Centre on Drug Policy Evaluation, MAP Centre for Urban Health Solutions, St. Michael’s Hospital, Toronto, ON Canada; 4 Research Centre, Centre Hospitalier de l’Université de Montréal (CRCHUM), Montréal, QC Canada; 5 Department of Psychiatry and Addictology, Faculty of Medicine, Université de Montréal, Montréal, QC Canada; 6 Modeling in Health Research Center, Institute for Futures Studies in Health, Kerman University of Medical Sciences, Kerman, Iran; 7 Dalla Lana School of Public Health, University of Toronto, Toronto, ON Canada; 8 Department of Epidemiology, New York University School of Global Public Health, New York, NY, USA

**Keywords:** People who inject drugs, Injection cessation, Relapse to injection, Retrospective cohort study, HIV, Hepatitis C

## Abstract

**Background:**

Drug injection is a major health-related problem worldwide. Injection cessation and relapse to injection could significantly alter the risk of HIV and hepatitis C virus (HCV) among people who inject drugs (PWID). This study aimed to estimate the rate of injection cessation and relapse to injection among PWID in Iran.

**Methods:**

This cohort study was conducted from 2018 to 2021 in the cities of Kerman and Tehran. Using a respondent-driven sampling (RDS) approach, 118 PWID with a history of injection in the last six months and negative HIV and HCV tests were recruited. Follow-up visits occurred every three months over a period of one year. Participants were interviewed and tested for HIV and HCV using rapid tests. Injection cessation was defined as the no injection of any type of drugs in the last three months. Relapse to injection was defined as re-initiating drug injection among those who had ceased injection. Two separate Cox regression models were applied, and an adjusted hazard ratio (aHR) with a 95% confidence interval (CI) were measured to assess the factors associated with each outcome.

**Results:**

The rate of injection cessation was 26.1 (95% CI: 21.3, 32.0) per 100 person-years, and the rate of relapse to injection was 32.7 (95% CI: 24.7, 43.2) per 100 person-years. At the baseline interview, 39.8% (n = 47) of participants reported injection cessation in the past three months before the interview. In the multivariable Cox regression analysis, the rate of relapse to injection was greater among women (aHR = 1.58; 95% CI: 1.01, 2.52), and those with higher monthly income (aHR = 1.63; 95% CI: 1.03, 2.59). However, there was no significant variable that predicted injection cessation.

**Conclusion:**

Injection cessation was common among PWID in Iran, however, one-third relapsed to injection shortly after cessation. Harm reduction programs should include comprehensive strategies to reduce the probability of relapse among PWID who achieve injection cessation.

## Introduction

The prevalence of human immunodeficiency virus (HIV) and hepatitis C virus (HCV) is notably high among people who inject drugs (PWID). In 2017, global estimates suggested that 17.8% of PWID were living with HIV and 52.3% were diagnosed with HCV antibody (HCV-Ab) [1]. Despite the limited quality and quantity of HIV data in the Eastern Mediterranean Region (EMR) [2], Iran has the highest prevalence of HIV among PWID in this region [3]. According to the results of the latest national bio-behavioural surveillance survey (BBSS) in 2020, the prevalence of HIV and HCV among PWID in Iran was 3.5% (95% confidence intervals [CI]: 2.9, 4.3) [4] and 26.0% (95% CI: 24.4, 27.7), respectively [5]. High-risk injecting behaviours, such as shared injection are considered the main mode of HIV and HCV transmission among PWID [6, 7], and in the BBSS in 2020, 87.9% of PWID reported using needle exchange programs in the last 12 months [5]. Several factors may contribute to the prevention of high-risk injecting behaviours, including practices, such as utilizing needle exchange programs and periods of injection cessation.

Injection cessation (i.e., stopping drug injection for a specific period), and relapse to injection (i.e., reinitiating drug injection after a period of injection cessation) are recognized as two important behaviours among PWID due to their association with a heavy burden of morbidity and mortality in this population [8]. Multiple episodes of injection cessation and relapse to injection have been documented as recurrent patterns within the cycle of injecting behaviours [9, 10]. There is a large body of evidence suggesting that injection cessation even within short periods (e.g., three months constantly) diminishes the risk of injecting-related adverse outcomes, such as HIV and HCV transmission and fatal overdose [8, 9, 11, 12]. For example, the hazard ratio of HCV infection among a cohort of PWID in a Canadian setting was 0.24 for those who ceased injection for three months [9].

Individual factors (e.g., being young and male), drug-related behaviours (e.g., no history of recent benzodiazepine use), socioeconomic status (e.g., unemployment and having limited social support), and environmental factors (e.g., homelessness and limited access to social services) have been reported to be associated with injection cessation and relapse into the injection [13–16]. Identifying factors that could prolong the injection cessation periods and decrease the tendency to relapse to injection among PWID could help develop practical interventions to decrease the probability of HIV and HCV transmission in PWID [13].

Although PWID is the most at-risk population for HIV and HCV in Iran as a result of unsafe injection-related behaviours, no study has assessed the rate of injection cessation and relapse to injection among PWID in Iran. This study aimed to assess injection cessation and relapse and the related factors in a cohort of PWID in two large cities in Iran. We also estimated the incidence of HIV and HCV among this cohort as the secondary outcome.

## Methods

### Study design

This study was the cohort phase of the Rostam study. The Rostam study had three phases: A cross-sectional bio-behavioural survey of HIV and HCV among PWID, a prospective cohort study among PWID without HIV and HCV infections, and a trial of an HCV model of care among PWID [17]. The cohort phase of this study was conducted from July 2018 to February 2021 in Kerman (southeast) and Tehran (the capital, central north). Participants were recruited for the study through a respondent-driven sampling (RDS) approach. Participants were eligible if they were ≥ 18 years old, self-reported drug injection at least once within the past six months, reported residency in the study cities, tested non-reactive for both HIV-Ab and HCV-Ab (or HCV RNA-negative for individuals with reactive HCV-Ab test) at the baseline visit, provided verbal consent to participate in the study, and had a valid referral RDS coupon of the study.

### Recruitment process

To initiate the recruitment process, five individuals from each city who were well-respected PWID within their communities and had large social networks of peers were selected as the study’s seeds. Each seed received three RDS referral coupons, which were valid for three weeks. The study team trained the seeds to refer their peers to the study. Each PWID who was eligible and participated in the study received three referral RDS coupons and the process was repeated until the required sample size was recruited. Every participant received ~ 2 United States Dollars (USD) as an incentive for completing the survey questionnaire and HIV and HCV tests. They also received ~ 1 USD for each successfully referred eligible individual in the study.

### Follow-up visits

After the baseline interview, a date for the next visit was set for each participant. A midterm visit was set at 45 days after the main visits, including only HIV and HCV blood tests. The main follow-up visits were set at three months after each main visit. The main follow-up visit included a face-to-face interview, and HIV and HCV tests. The follow-up process continued for up to 12 months (4 different interviews within 3 months period) for each participant. If any participants missed the scheduled visit for two weeks or more, the visit was considered as a loss to follow-up [18]. Moreover, the participants received 1 USD and 1.5 USD for each midterm visit and main follow-up visit, respectively.

### Data collection instruments, HIV, and HCV testing

A standard questionnaire was used to collect PWID’s characteristics and behaviours. The questionnaire included socio-demographic characteristics and different behaviours within the last three months, including non-injection drug use history, drug injection history, sexual behaviours history, HIV testing, HIV knowledge, and harm reduction service utilization. A gender-matched trained interviewer conducted the face-to-face interview in a private room inside the facility via a tablet-based questionnaire. After the interview, participants were tested by a rapid HIV test (SD-Bioline, South Korea) followed by a confirmatory test (Unigold HIV test), for those who had a reactive rapid HIV test result. PWID who were positive for both HIV tests were considered HIV positive in this study. Also, an HCV-Ab rapid test (SD-Bioline, South Korea) was done for each participant and those with reactive HCV-Ab rapid test were also tested for HCV RNA to determine the presence of HCV infection.

### Outcomes definition: injection cessation and relapse

The cessation of injection was defined as self-reported no history of injecting any type of drugs within the past three months. The relapse to injection was defined as self-reported reinitiation of drug injection after reporting an injection cessation episode in the previous interviews.

### Covariates

The covariates of interest included socio-demographic and substance-use-related behavioural variables. The socio-demographic variables included age (per year), gender (man, woman), education (high school or above, less than high school), monthly income (< 100 USD, ≥ 100 USD), living with spouse or partner (yes, no), and incarceration history in the last three months (yes, no). Substance use-related variables included length of injection career (≤ one year, more than one to five years, > five years), history of overdose in the last three months (yes, no), receipt of free needles/syringes in the last three months (yes, no), receipt of opioid agonist therapy (OAT) in the last three months (yes, no), cannabis use (yes, no), non-injection heroin use (yes, no), non-injection methamphetamine use (yes, no), injection heroin use (yes, no), injection methamphetamine use (yes, no), history of receptive shared injection (yes, no), history of receptive shared injection equipment (yes, no), history of HIV testing (ever, never), and history of HCV testing (ever, never). The time frame for all substance use-related practices was the last three months.

### Statistical analysis

Cox proportional hazard model was used to determine the factors related to injection cessation and relapse. Time to injection cessation was defined as the time from the study initiation to the occurrence of a cessation episode. Similarly, time to relapse was defined as the duration from cessation to the occurrence of a drug injection episode. The Andersen-Gill (AG) model [19], an extension of the Cox model, was employed to apply the counting process approach in order to calculate hazard ratios (HR). The person-time was measured up to the date of the interview, including the three months before the interview date. To account for the correlation among outcomes within the same subject, robust estimation was utilized to derive variance estimators. The Grambsch-Therneau test, which relies on scaled Schoenfeld residuals, was used to assess the proportional hazard assumption. HR with 95% CI was measured for each covariate and the covariates with P-value < 0.2 [19] were imported to the multivariable Cox regression. As using a weighted regression model for analyzing RDS data could result in inflated type-I error, poor parameter coverage, and biased results, the unweighted analysis was used to conduct the primary regression model [20]. The backward elimination method was used to simplify the final model and the covariates with a P-value < 0.05 were considered statistically significant. Stata (version 17) was used for all of the analyses.

## Results

### Baseline characteristics

**Fig. 1:**
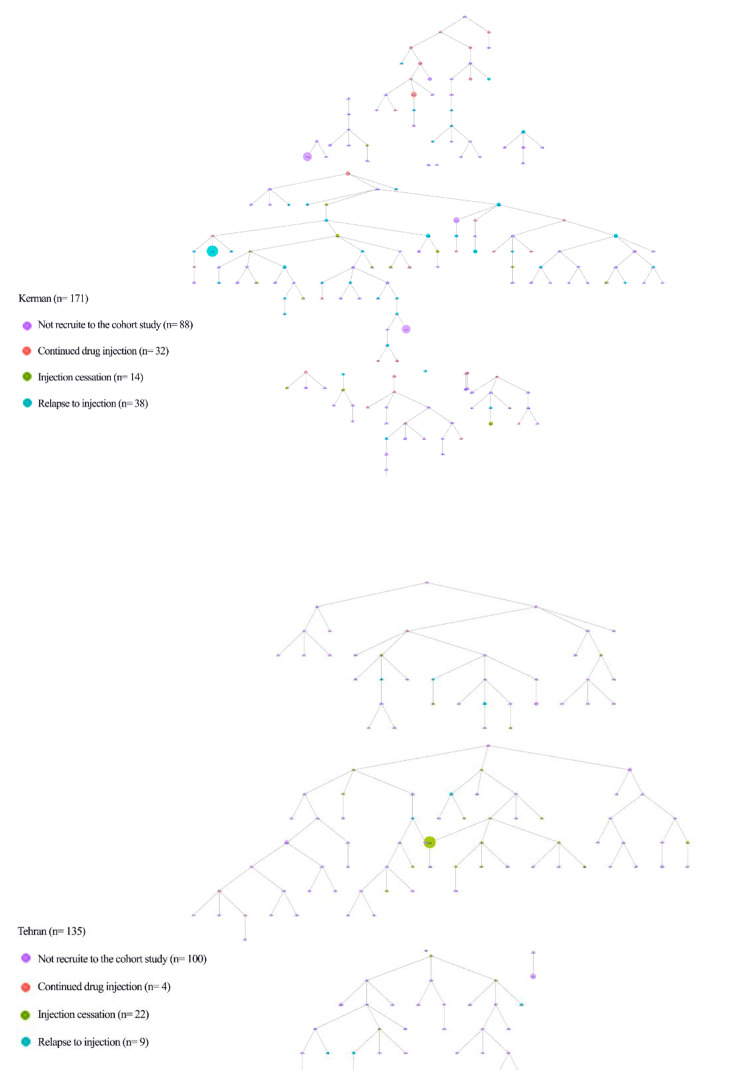
The RDS referral chain of people who inject drugs in Kerman and Tehran, Iran, 2018–2021

**Fig. 2:**
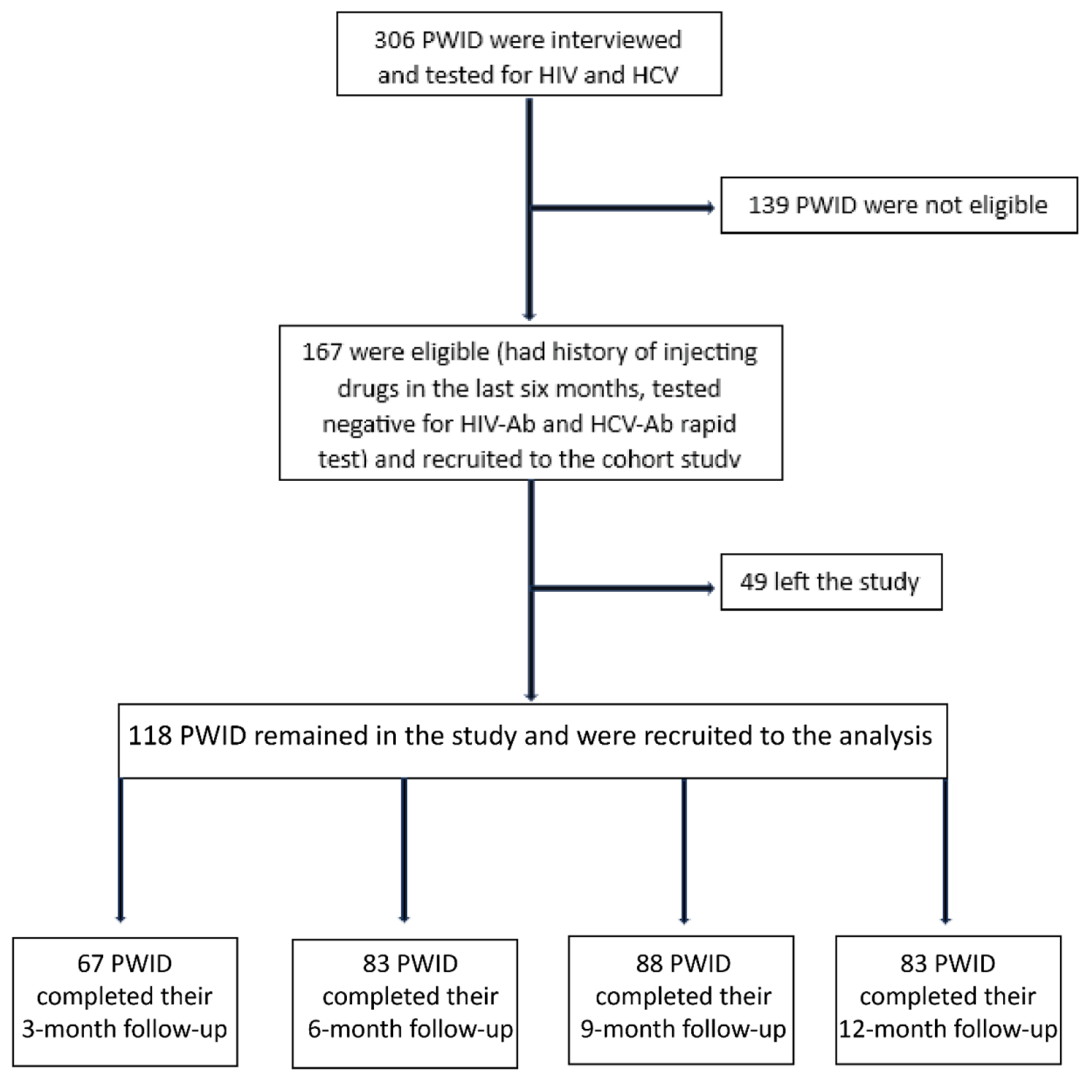
The flow chart of people who inject drugs recruitment to the cohort study of Kerman and Tehran, Iran, 2018–2021

Overall, 306 PWID were recruited through RDS referral chains at the baseline interview and 167 of them were eligible and invited to the cohort phase of the study (Fig. 1). Only, 118 (70.6%) participants returned to the study sites for at least one follow-up visit who were recruited to the analysis (Fig. 2). The mean age (standard deviation [SD]) of participants at the baseline visit was 43.3 (10.8) years old and the majority (n = 106, 89.8%) were men (Table 1). The majority of participants (n = 73, 67.6%) had injected drugs for more than five years, and (n = 57, 72.2%) received free needles/syringes within the last three months. The prevalence of self-reported lifetime HIV and HCV testing among the participants was 47.3% (n = 53) and 11.4% (n = 12), respectively.

**Table 1:** Baseline characteristics of the people who injected drugs included in the cohorts of Kerman and Tehran, Iran, 2018–2021 (N = 118)

Variable	Kerman (n = 83)	Tehran (n = 35)	Total (n = 118)	P-value
	N (%)	N (%)	N (%)	
**Age**				
Mean (standard deviation)	40.2 (9.2)	50.4 (11.4)	43.3 (10.8)	< 0.001
**Gender**				
Women	11 (13.3)	1 (2.9)	12 (10.2)	0.088
Men	72 (86.7)	34 (97.1)	106 (89.8)	
**Education**				
High school or above	30 (36.1)	14 (41.2)	44 (37.6)	0.621
Less than high school	53 (63.9)	20 (58.8)	73 (62.4)	
**Monthly income**				
≥ 100 USD	55 (67.9)	14 (53.8)	69 (64.5)	0.046
< 100 USD	26 (32.1)	12 (46.2)	38 (35.5)	
**Living with spouse or partner**				
Yes	8 (9.6)	4 (11.8)	12 (10.2)	0.731
No	75 (90.4)	30 (88.2)	105 (89.8)	
**Incarceration (L3M** ^**a**^ **)**				
Yes	14 (16.9)	0 (0.0)	14 (11.9)	0.010
No	69 (83.1)	35 (100.0)	104 (88.1)	
**Length of injection career**				
≤ 1 years	8 (10.7)	4 (12.1)	12 (11.1)	0.585
2–5 years	18 (24.0)	5 (15.2)	23 (21.3)	
> 5 years	49 (65.3)	24 (72.7)	73 (67.6)	
**Received free needle/ syringes (L3M** ^**a**^ **)**				
Yes	46 (80.7)	11 (50.0)	57 (72.2)	0.006
No	11 (19.3)	11 (50.0)	22 (27.8)	
**Receipt OAT (L3M** ^**a**^ **)**				
Yes	73 (88.0)	7 (20.0)	80 (67.8)	< 0.001
No	10 (12.0)	28 (80.0)	38 (32.2)	
**Non-injection heroin use (L3M** ^**a**^ **)**				
Yes	76 (91.6)	22 (62.9)	98 (83.0)	< 0.001
No	7 (8.4)	13 (37.1)	20 (17.0)	
**Non-injection methamphetamine use (L3M** ^**a**^ **)**				
Yes	75 (90.4)	11 (31.4)	86 (72.9)	< 0.001
No	8 (9.6)	24 (68.6)	32 (27.1)	
**Injection heroin use (L3M** ^**a**^ **)**				
Yes	48 (87.3)	12 (85.7)	60 (87.0)	0.877
No	7 (12.7)	2 (14.3)	9 (13.0)	
**Injection methamphetamine use (L3M** ^**a**^ **)**				
Yes	8 (9.9)	24 (77.4)	32 (28.6)	< 0.001
No	73 (90.1)	7 (22.6)	80 (71.4)	
**Receptive shared injection (L3M** ^**a**^ **)**				
Yes	3 (5.4)	2 (12.5)	5 (7.0)	0.332
No	52 (94.6)	14 (87.5)	66 (93.0)	
**Receptive shared injection equipment (L3M** ^**a**^ **)**				
Yes	6 (11.1)	3 (18.7)	9 (12.9)	0.423
No	48 (88.9)	13 (81.3)	61 (87.1)	
**History of HIV test**				
Ever	40 (50.6)	13 (39.4)	53 (47.3)	0.277
Never	39 (49.4)	20 (60.6)	59 (52.7)	
**History of HCV test**				
Ever	6 (8.3)	6 (18.2)	12 (11.4)	0.141
Never	66 (91.7)	27 (81.8)	93 (88.6)	

a: Last 3 months

### Loss to follow up

Among the participants who were recruited for the analysis, 31.4% (n = 37) had no loss to follow-up. Among the participants who missed at least one visit, 3.4%, 7.6%, and 18.6% were lost to follow-up after the first, second, and third visit, respectively, and did not return to the study. Overall, 94.1% of participants reported receiving OAT during the follow up. Furthermore, the only distinguishing factor between participants with loss to follow-up and those without was their history of incarceration within the last three months.

### Injection cessation and relapse to injection

**Fig. 3:**
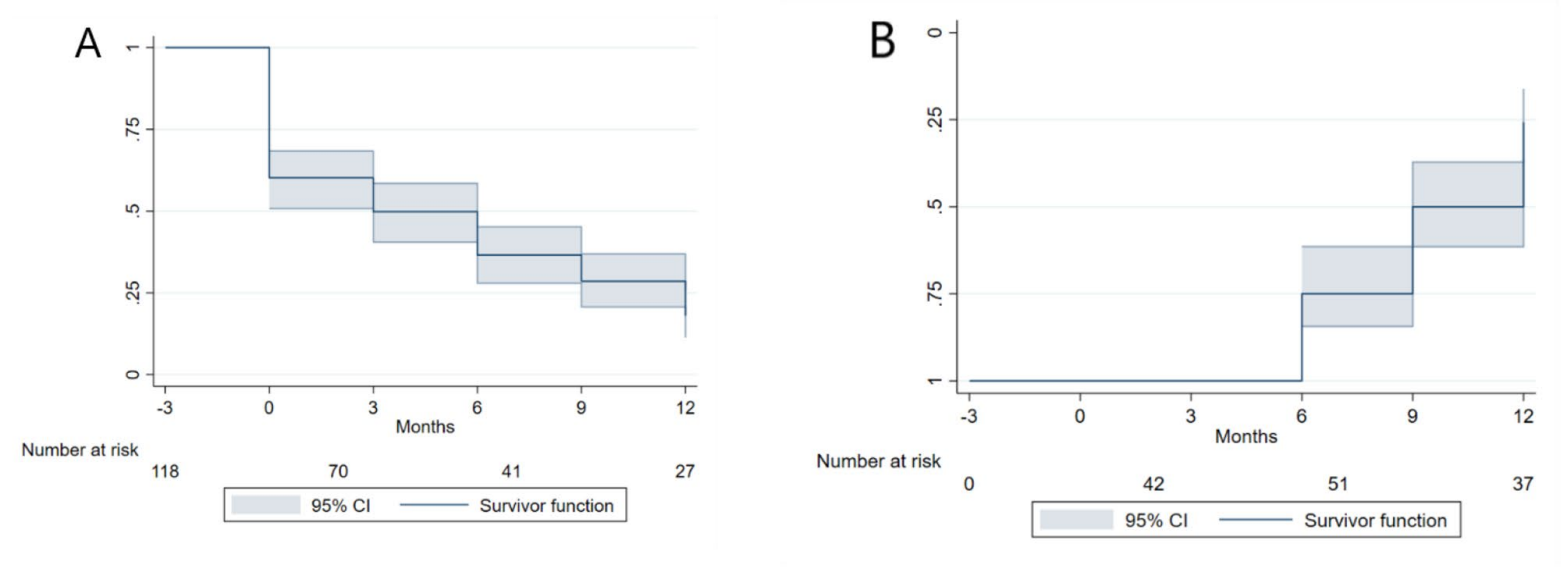
The Kaplan-Meier estimate of time of injection cessation (A) and relapse to injection (B) among the PWID in Kerman and Tehran, Iran, 2018–2021

Out of 118 participants with at least one follow-up visit, 83 (70.3%) participants reported injection cessation at least once during the study. The overall incidence rate of injection cessation was 26.1 (95% CI: 21.3, 32.0) per 100 person-years. Of those with at least one injection cessation episode, 47 (56.6%) reported relapse to injection during the study. The incidence rate of relapse to injection was 32.7 (95% CI: 24.7, 43.2) per 100 person-years (Fig. 3).

### Correlates of injection cessation incidence

According to the results of the bivariable and multivariable analysis, living in Tehran was significantly and positively associated with injection cessation (cHR = 1.50; 95% CI: 1.09, 2.06) (Table 2).

**Table 2:** Estimated association between covariates and hazards of injection cessation and relapse to injection among the people who inject drugs in Kerman and Tehran, Iran, 2018–2021

Variable	Injection cessation				Relapse to injection		
	cHR^a^	95% CI^b^	P-value		cHR^a^	95% CI^b^	P-value
**City**							
Tehran	1.50	1.09, 2.06	0.013		0.61	0.36, 1.05	0.074
Kerman	Ref				Ref		
**Age**	1.00	0.98, 1.02	0.979		0.99	0.97, 1.01	0.588
**Gender**							
Women	1.13	0.67, 1.88	0.647		1.56	1.01, 2.41	0.045
Men	Ref				Ref		
**Education**							
High school or above	1.06	0.75, 1.48	0.742		0.76	0.46, 1.14	0.165
Less than high school	Ref				Ref		
**Monthly income**							
≥ 100 USD	1.35	0.95, 1.91	0.095		1.62	1.02, 2.58	0.043
< 100 USD	Ref				Ref		
**Living with spouse or partner**							
Yes	1.27	0.78, 2.07	0.341		1.67	0.96, 2.89	0.067
No	Ref				Ref		
**Incarceration (L3M** ^*^ **)**							
Yes	1.19	0.76, 1.87	0.434		0.71	0.30, 1.67	0.431
No	Ref				Ref		
**Length of injection career**							
≤ 1 years	1.42	0.82, 2.45	0.213		1.48	0.91, 2.40	0.112
2–5 years	1.21	0.76, 1.91	0.425		1.64	1.04, 2.59	0.034
> 5 years	Ref				Ref		
**Received free needles/syringes (L3M** ^*^ **)**							
Yes	1.13	0.75, 1.68	0.553		0.75	0.44, 1.29	0.303
No	Ref				Ref		
**Receipt OAT (L3M** ^*^ **)**							
Yes	0.91	0.63, 1.32	0.627		0.96	0.48, 1.89	0.903
No	Ref				Ref		
**Non-injection heroin use (L3M** ^**c**^ **)**							
No	1.11	0.66, 1.87	0.674		0.79	0.33, 1.87	0.597
Yes	Ref				Ref		
**Non-injection methamphetamine use (L3M** ^**c**^ **)**							
No	1.27	0.82, 1.98	0.285		1.14	0.60, 2.15	0.410
Yes	Ref				Ref		
**History of HIV test**							
Ever	0.90	0.60, 1.33	0.597		0.94	0.37, 2.43	0.905
Never	Ref				Ref		
**History of HCV test**							
Ever	0.97	0.57, 1.64	0.905		1.84	0.93, 3.63	0.079
Never	Ref				Ref		

a: Crude hazard ratio

b: Confidence interval

c: Last three months

### Correlates of relapse to injection

Based on the results of the bivariable analysis, the hazard of relapse to injection was significantly higher among women (cHR = 1.56; 95% CI: 1.01, 2.41), and PWID who had a monthly income of > 100 USD (cHR = 1.62; 95% CI: 1.02, 2.58), and had 2–5 years of injection career length (cHR = 1.64; 95% CI: 1.04, 2.59) (Table 2). The results of the multivariable model illustrated that the adjusted hazard ratio (aHR) for relapse to injection was higher among women (aHR = 1.58; 95% CI: 1.01, 2.52) and those who had a higher monthly income (aHR = 1.63; 95% CI: 1.03, 2.59) (Table 3).

**Table 3:** Multivariable Cox proportional hazard regression models of factors associated with incidence of relapse to injection among the people who inject drugs in Kerman and Tehran, Iran, 2018–2021

	Relapse to injection		
	aHR^a^	95% CI^b^	P-value
**Gender**			
Women	1.58	1.01, 2.52	0.048
Men	Ref		
**Monthly income**			
≥ 100 USD	1.63	1.03, 2.59	0.038
< 100 USD	Ref		

a: Adjusted hazard ratio

b: Confidence interval

### HIV and HCV incidence

None of the participants had reactive test results for HIV or HCV during the study, and the incidence rate for both HIV or HCV was zero.

## Discussion

Our cohort study showed that about two-thirds of the participants had at least one episode of injection cessation lasting for three months or more. Furthermore, more than half of PWID with a history of injection cessation had a history of relapse to injection during the follow-up period. Women and individuals with a higher monthly income were about 1.6 times more likely to relapse to injection after at least one episode of injection cessation. Moreover, no new infections of HIV and HCV were detected throughout the study.

We found that about two-thirds of PWID reported an injection cessation in the follow-up period, corresponding to an incidence rate of around 26 per 100 person-years. The proportion of PWID who experienced injection cessation at least once in this cohort was higher compared to similar studies conducted elsewhere. For example, previous studies in Australia (5.4 per 100 person-years) [15], Mexico (7.3 per 100 person-years) [13], United States, California, San Francisco (16.4 per 100 person-years) [21] and, Baltimore (7.6 per 100 person-years) have reported lower rates of injection cessation [22]. The higher incidence rate of injection cessation in Iran could be attributed to our definition of injection cessation that covered a shorter period (three months) compared to other studies. The other justifiable reason for higher rate of injection cessation in our study could be the high prevalence of OAT uptake in our study. Almost, all participants in our study had received OAT at least once during the study. It is well stablished that providing OAT services, such as methadone or buprenorphine could be a practical strategy to facilitate injection cessation [23].

The incidence rate for relapse to injection was around 33 per 100 person-years among those with a history of injection cessation. This finding was comparable with findings of other settings, such as India (19.7 per 100 person-years) [8] and United States, California (55.5 per 100 person-years) [21]. Injection cessation and relapse to injection have been suggested as a cycle in the drug injection process [9]. Future qualitative studies of PWID in Iran should investigate the possible reasons for such a high relapse rate. The high rate of relapse to injection in this cohort could be attributed to the absence of comprehensive programs aimed at preventing relapse to injection among PWID who have chosen to cease injection. Introducing educational initiatives that emphasize practical strategies, such as engaging in self-talk to consider the adverse effects of drug injection, avoiding environments and individuals that may heighten the inclination to inject, participating in available programs like Narcotics Anonymous, and exploring alternative modes of drug administration, could significantly extend the period of injection cessation among PWID who have successfully stopped injecting [24].

The absence of HIV and HCV new infections in our study may be attributed to several factors, including the relatively short follow-up period, the implementation of a comprehensive harm reduction program in the country, and a high rate of injection cessation observed among the participants. Previous surveys have found that even short periods of injection cessation could significantly reduce the risk of HIV and HCV acquisition [9] which could explain no newly diagnosed cases of HIV and HCV among the participants in our study. Moreover, a history of injection cessation in the past could increase its likelihood over time which promotes the risk reduction for these infections [10, 16]. Furthermore, at the baseline interview, approximately two-thirds of the participants reported having received OAT, and three-fourths reported having accessed free needle and syringe services within the last three months. These findings indicate a high coverage of harm reduction services among PWID in this study. It is widely recognized that harm reduction programs, including needle and syringe distribution, play a crucial role in effectively reducing HIV and HCV transmission among PWID [25–28].

### Limitation

We acknowledge our study’s limitations. First, due to the COVID-19 pandemic in Iran and lockdown policies, the study team had to terminate the cohort study and we were unable to determine potential long-term changes in PWID’s behaviours. Second, injection cessation and relapse to injection were measured based on self-report, which introduces the possibility of recall bias, reporting bias, and social desirability bias. However, the study team made efforts to mitigate recall bias by conducting short, frequent interviews as well as employing local and experienced interviewers. Third, this study was conducted only in two cities among a small sample of PWID and the findings may not be generalizable to the total PWID population of Iran. Fourth, the participants were recruited by the RDS sampling method. It is important to note that participants recruited through RDS coupons may share similarities with their respective seeds in terms of injection-related behaviours. However, it is worth mentioning that by limiting the number of seeds in each city to increase the length of recruitment chains, the similarity between the participants and the seeds should have decreased. Fifth, the study had limited participation from women, and therefore, the findings cannot be generalized to the broader population of women who inject drugs. Further investigation specifically focusing on women who inject drugs is warranted.

## Conclusion

Injection cessation (26 per 100 person-years) and relapse (33 per 100 person-years) are common among PWID in Iran. The findings underscore the challenges and complexities that individuals encounter when attempting to break the cycle of injection drug use. Notably, the study revealed a significant rate of relapse following cessation. Moreover, the absence of new HIV and HCV infections detected throughout the study period is encouraging and might imply that harm reduction and preventive measures in place may be effectively curbing the transmission of these bloodborne diseases among PWID in Iran. These findings can inform policy and healthcare initiatives aimed at reducing the harm associated with injection drug use and supporting individuals on their journey towards reduced harms and potential recovery.

## Data Availability

The data of the current study is available.

## Abbreviations

PWID: People who inject drugs
RDS: Respondent driven sampling
HCV: Hepatitis C virus
HR: Hazard ratio
cHR: Crude hazard ratio
aHR: Adjusted hazard ratio
CI: Confidence interval
HCV-Ab: HCV antibody
EMR: Eastern Mediterranean Region
HCV RNA: Hepatitis C virus RNA
USD: United states dollar
OAT: Opioid agonist therapy
AG: Andersen-Gill
SD: Standard deviation
